# Ubiquitin is double-phosphorylated by PINK1 for enhanced pH-sensitivity of conformational switch

**DOI:** 10.1007/s13238-019-0644-x

**Published:** 2019-07-05

**Authors:** Shang-Xiang Ye, Zhou Gong, Ju Yang, Yu-Xin An, Zhu Liu, Qun Zhao, Ewen Lescop, Xu Dong, Chun Tang

**Affiliations:** 1 Wuhan National Laboratory for Optoelectronics, Huazhong University of Science and Technology, Wuhan, 430074 China; 2 CAS Key Laboratory of Magnetic Resonance in Biological Systems, State Key Laboratory of Magnetic Resonance and Atomic Molecular Physics, National Center for Magnetic Resonance at Wuhan, Wuhan Institute of Physics and Mathematics of the Chinese Academy of Sciences, Wuhan, 430071 China; 3 Institut de Chimie des Substances Naturelles, CNRS UPR 2301, Univ Paris-Sud, Université Paris-Saclay, 91198 Gif-sur-Yvette cedex, France; 4 CAS Key Laboratory of Separation Sciences for Analytical Chemistry, Dalian Institute of Chemical Physics of the Chinese Academy of Sciences, Dalian, 116023 China

**Dear Editor,**

Multisite phosphorylation is observed in many signaling proteins, which confers the proteins new functions. A multisite phosphorylated protein can exhibit switch-like response to cellular stimuli, with the often-clustered phosphorylation sites either unphosphorylated or fully phosphorylated by the same kinase (Kapuy et al., 2009). Multisite phosphorylation has also been reported to promote the folding of an intrinsically disordered protein, and consequently modulate the binding affinity to other proteins (Bah et al., 2015). Here we show that multisite phosphorylation makes a protein a better pH sensor, promptly switching between alternative conformational states in response to pH change under physiological conditions.

Ubiquitin (Ub) is a 76-residue signaling protein in cells. Proteomics studies have shown that almost all serine/threonine residues in Ub can be phosphorylated (Swatek and Komander, 2016). However, the only Ub kinase identified to date is PINK1, which phosphorylates Ub at residue S65 (Kane et al., 2014; Koyano et al., 2014; Gladkova et al., 2017). PINK1 phosphorylation of Ub can activate PARKIN (Gladkova et al., 2018), an E3 ligase, which in turn leads to cell mitophagy and slows the progression of Parkinson’s disease. Thus, mutations of PINK1 are implicated in the early onset of Parkinson’s disease (Kane et al., 2014; Koyano et al., 2014). Here we report that PINK1 can also phosphorylate Ub at residue T66. We first identified a peptide from *in vitro* enzymatic assay that corresponds to the mass of both S65 and T66 phosphorylated (Fig. 1A). Upon co-transfection of Ub 7KR (mutating all seven lysines to arginines thus blocking Ub chain formation) and soluble PINK1 (Gao et al. 2016) to HEK293 cells, we also identified S65 and T66 double-phosphorylated peptide (Fig. 1B).

**Figure 1:**
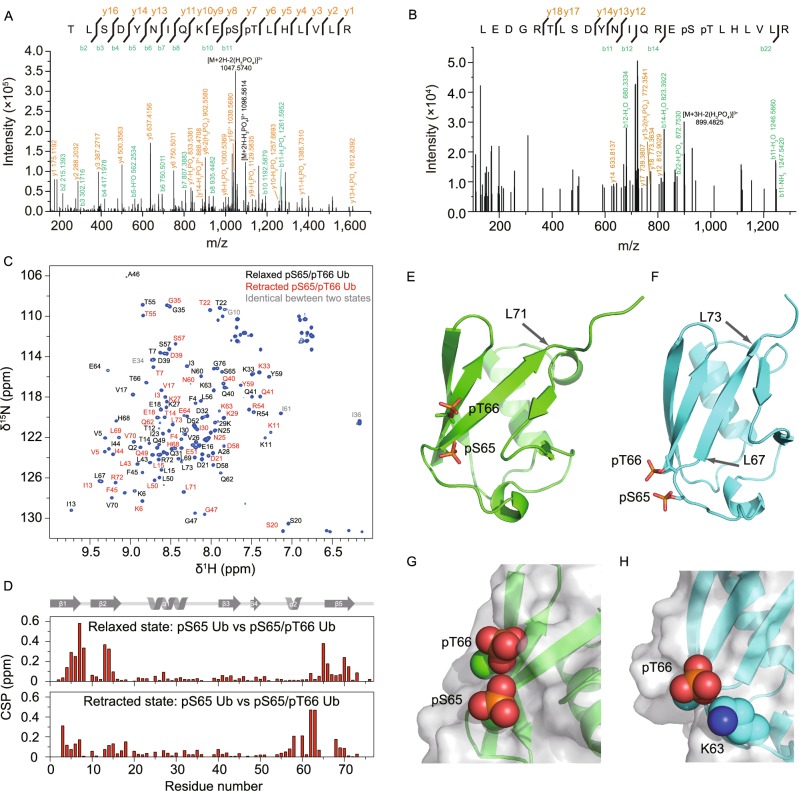
**Double phosphorylation of Ub by PINK1 at S65 and T66 sites**. (A) MS2 spectrum showing the peptide derived from Ub containing the two phosphorylated residues, with the Ub protein phosphorylated *in vitro*. (B) MS2 spectrum showing the double phosphorylated Ub peptide, with the Ub protein phosphorylated by PINK1 in cells. To avoid the formation of polyubiquitin and to simply analysis, Ub 7KR mutant was co-transfected with soluble PINK1 (residues 102–521) into HEK293 cells. (C) NMR analysis of pS65/pT66 Ub. 2D ^1^H-^15^N HSQC spectrum collected at 25 °C in pH 7.4 buffer on a 600 MHz spectrometer. The peaks can be assigned to a C-terminally relaxed state and retracted state undergoing slow exchange, with some peaks degenerate. (D) NMR chemical shift perturbations (CSPs) are relatively small, when comparing to pS65 single phosphorylated Ub in the same conformational state. The averaged CSP in ppm is calculated with the equation Δδ = [0.5 × ΔδH^2^ + 0.1 × ΔδN^2^]^1/2^, with ΔδH and ΔδN the CSPs in ^1^H and ^15^N dimensions, respectively. Ub secondary structure is indicated. (E and F) Solution structures of pS65/pT66 Ub in the C-terminally relaxed and retracted state, respectively. The retracted state mainly differs from the relaxed state in the register of the C-terminal β-strand (β5), moving up by 2 residues. (G) Close-up view of the phosphoryl groups in the relaxed state, which are partially buried. (H) Close-up view of residues pT66 and K63 in the retracted state. The proximity of pT66 and K63 suggests electrostatic interaction, which would explain the increased stability of Ub retracted state.

We used nuclear magnetic resonance (NMR) spectroscopy to characterize the structure of the recombinantly purified pS65/pT66 Ub protein. The 2D ^1^H-^15^N heteronuclear single quantum correlation (HSQC) spectrum of the double-phosphorylated Ub shows more than 120 peaks, nearly twice as much as expected for a 76-residue protein. The peaks can be assigned to two alternative conformational states (Fig. 1C). It has been previously shown that, S65 single-phosphorylated Ub exists in two conformational states, namely the C-terminally relaxed state and retracted state. The structure of pS65 Ub relaxed state is similar to that of unmodified Ub. The retracted state, however, differs from the relaxed state mainly in the position of the C-terminal β-strand (β5), which moves towards its N-terminal end by two residues (Dong et al., 2017). The two conformational states of pS65/pT66 Ub should also correspond to C-terminally relaxed and retracted states, respectively. Indeed, the chemical shift perturbations (CSPs) between pS65/pT66 Ub and pS65 Ub in same conformational state are smaller than the CSPs between the two proteins in different conformational states (Figs. 1D and S1). Additionally, residual dipolar coupling (RDC) data measured for backbone amide bond vectors have similar overall profiles for pS65/pT66 Ub and pS65 Ub in presumably the same conformational state (Fig. S2).

We prepared an Ub/S65A mutant, and found that the protein can also be phosphorylated by PINK1 (Fig. S3A). For the ^15^N-labeled pT66 Ub/S65A, we observed a single peak in the ^31^P spectrum for the phosphoryl group (Fig. S3B), and a single set of peaks in the 2D ^1^H-^15^N HSQC spectrum for backbone amide groups (Fig. S3C). The CSP profile of ^1^H-^15^N peaks between pT66 Ub/S65A and Ub/S65A is similar to that between pS65/pT66 Ub and pS65 Ub both in relaxed state. Moreover, the residues with large CSPs upon phosphorylation can be mostly mapped to around pT66 (Fig. S3E). Thus, pT66 Ub/S65A mutant should only exist in the C-terminally relaxed state, and T66 phosphorylation alone does not induce Ub conformational switch.

Refining against multiple types of experimental data, we determined the NMR structures for the two conformational states of pS65/pT66 Ub (Table S1). The root-mean-square (RMS) deviations for the backbone heavy atoms of pS65/pT66 Ub relaxed and retracted states are 0.32 ± 0.04 Å and 0.75 ± 0.09 Å, respectively (Fig. S4). The last β-strand of pS65/pT66 Ub encompasses residues 65–71 in the relaxed state, and residues 67–73 in the retracted state, thus accounting for the shift in hydrogen-bonding register. Comparing to the structures of pS65 Ub, the backbone RMS differences are 0.79 ± 0.06 Å and 1.81 ± 0.11 Å, for the C-terminally relaxed and retracted states, respectively (Fig. S5). The larger structural difference for the retracted state is consistent with the less good agreement for the backbone RDCs (Fig. S2).

The phosphoryl groups are positioned differently in the two conformational states of pS65/pT66 Ub. In the relaxed state, the phosphoryl groups are partially buried and close to each other (Fig. S4A). Many peaks are missing from the NMR spectra of the retracted state, and as a result, the structure is less well-defined, especially around the protruding loop preceding β5 (Fig. S4A). The local dynamics of the retracted state is probably resulted from the electrostatic repulsion between the adjacent phosphoryl groups. Nevertheless, with the β5 retracted, pT66 phosphoryl group can electrostatically interact with K63 sidechain (Fig. 1H). Indeed, the distance between the oxygen atoms of pT66 phosphoryl group and K63 sidechain amine group is shorter in the retracted state, albeit with a broader distribution, than that in the relaxed state (Fig. S6).

To understand how the multisite phosphorylation impacts the energy landscape of Ub, we evaluated the relative abundance of the C-terminally relaxed and retracted states of pS65/pT66 Ub. The two states are almost equally populated at pH 7.4. At pH < 7.4, the HSQC peak intensities for the relaxed state of pS65/pT66 Ub are higher than those for the retracted state (Fig. S7), meaning that the relaxed state is more populated. Quantitation of HSQC peak intensities is problematic at basic pH owing to water exchange. Therefore, we evaluated the ^31^P peak volumes for the two phosphoryl groups of pS65/pT66 Ub, which can give more accurate quantitation of the relative populations. A total of four ^31^P peaks were observed, with two assigned to pS65 and two to pT66 in either relaxed state or retracted state (Fig. 2A). Judging from the ^31^P peak volume, when the pH increases, the population of the C-terminally retracted state of pS65/pT66 Ub increases, reaching ~80% population at pH 8.0. In comparison, the retracted state of the single-phosphorylated pS65 Ub is only ~60% populated at pH 8.0 (Dong et al., 2017). Moreover, the population profiles are similar for pS65 and pT66 (Fig. 2B), meaning that the two phosphoryl groups experience the same conformational switch in response to pH change. The population profiles of relaxed and retracted states of pS65/pT66 Ub can be globally fit to a Boltzmann sigmoidal function. Excellent fit was obtained with an interconversion slope of 0.63 ± 0.07 (Fig. 2B, red and blue curves). In comparison, fitting the population profile of single-phosphorylated pS65 Ub yields a slope of 0.35 ± 0.03 (Fig. 2B, gray curves). The steeper transition for the pS65/pT66 Ub means that the presence of the additional T66 phosphoryl group enhances the pH-sensitivity of Ub conformational switch, mainly by stabilizing the C-terminally retracted state at slightly basic pH.

**Figure 2:**
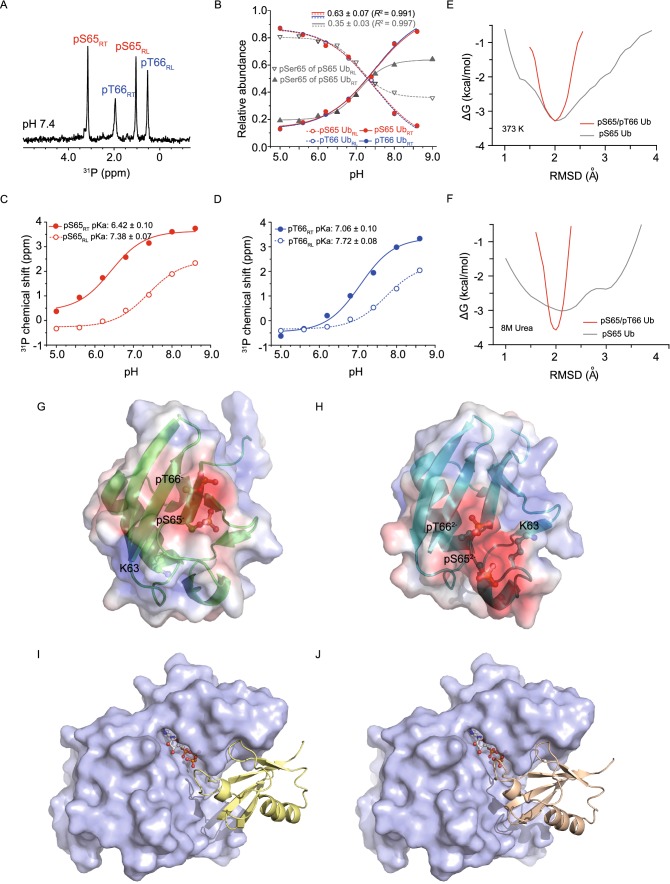
**The pS65/pT66 Ub switches between relaxed and retracted states in response to small change in pH**. (A) Four peaks are observed in the 1D ^31^P spectrum, and can be assigned to the phosphoryl groups of pS65 and pT66 in retracted state (RT) and relaxed state (RL), respectively. (B) Fitting the relative populations of retracted/relaxed states of pS65/pT66 Ub (red and blue lines) and pS65 Ub (gray lines) as a function of pH, using Boltzmann sigmoid function. The slope indicates the steepness of the transition between the two conformational states—the slope of the double phosphorylated Ub is almost twice as large as that of the single phosphorylated Ub. (C and D) Fitting the chemical shift values of the four ^31^P peaks, as a function of pH, yields four pKa values. (E and F) T66 phosphorylation helps to stabilize the retracted state. Reconstructed free energy landscape of the unfolding processes of pS65/pT66 Ub and pS65 Ub retracted states under heat (373 K) and 8 mol/L urea denaturing conditions, respectively. (G and H) Electrostatic surfaces of pS65/pT66 Ub in relaxed and retracted states, respectively, plotted at ±3kBT scale. With the phosphoryl groups protonated, the relaxed state of pS65/pT66 Ub has fewer overall negative charges than the retracted state. (I and J) Possible mechanism for double phosphorylation by PINK1, in which Ub undergoes rocking movement when binding to PINK1 between N- and C-lobes. T66 phosphorylation may be facilitated when Ub adopts a retracted-state conformation, with the loop preceding β5 further protruded (I).

To uncover the driving force for Ub conformational switch, we measured the pKa values of the phosphoryl groups based on the ^31^P chemical shift values. When the pH increases, all four ^31^P peaks shift downfield. Fitting the chemical shift values versus the pH yields the pKa values of 7.38 ± 0.07 and 7.72 ± 0.08 for pS65 and pT66 in the relaxed state, and 6.42 ± 0.10 and 7.06 ± 0.10 for pS65 and pT66 in the retracted state (Fig. 2C and 2D). In comparison, the pKa value for the phosphorylated serine in a random-coil peptide is 5.96 (Bienkiewicz and Lumb, 1999), while the pKa values for pS65 in the single-phosphorylated Ub are 7.21 in the relaxed state and 5.83 in the retracted state (Dong et al., 2017). Thus, with the additional phosphorylation at T66 in pS65/pT66 Ub, the pKa value for pS65 phosphoryl group is further elevated. The pKa value for the phosphorylated threonine in a random-coil peptide is 6.30 (Bienkiewicz and Lumb, 1999), and is 7.14 ± 0.08 for pT66 in Ub/S65A Ub (Fig. S3D). Thus, the pKa value is also elevated for pT66 phosphoryl group, especially in the relaxed state of pS65/pT66 Ub.

The higher pKa values indicate that the phosphoryl groups are more likely protonated at neutral pH, which can be explained by the partial burial of the charged phosphoryl groups in the relaxed state (Fig. 1G). Moreover, the close proximity of the two phosphoryl groups in the relaxed state, each carrying −2 formal charge if fully deprotonated, also makes the protonation necessary. At a pH value above the pKa values for the phosphoryl groups in the relaxed state, the phosphoryl groups become deprotonated, and as a result, the relaxed state would be destabilized. Thus, a more stable retracted state is favored to accommodate the deprotonated phosphoryl groups. Conversely, lowering the pH and increasing the proton concentration can switch the conformation of pS65/pT66 Ub from the retracted state to the relaxed state, as the protonated phosphoryl groups can be readily buried. Such a (de)protonation-triggered conformational switch mechanism also explains why Boltzmann sigmoid function is suitable to fit the population curves for the two conformational states. The Boltzmann sigmoid function has been used to describe the coupling between transferred ions and conformational switch of voltage-gated ion channels, with the slope proportional to the total charges displaced (Schoppa et al., 1992). Here, the interconversion slopes are almost twice as steep for the double-phosphorylated Ub than for the single-phosphorylated Ub (Fig. 2B), which supports the mechanism that two protons are transferred upon Ub conformational switch.

To further assess the energetic landscape of pS65/pT66 Ub, we measured the melting temperature for the double-phosphorylated Ub, which was found higher than that of the single-phosphorylated Ub (Fig. S8). Since the increased thermal stability can be attributed to both relaxed and retracted states, we performed MD simulations for pS65/pT66 Ub in the two conformational states under different types of denaturing conditions. The simulations showed that, the structure of deprotonated retracted state of pS65/pT66 Ub is more stable under both heat and urea denaturing conditions than that of pS65 Ub (Fig. 2E and 2F), while the structure of protonated relaxed state of pS65/pT66 Ub has similar stability to that of pS65 Ub (Fig. S9). Together, the additional T66 phosphorylation helps to stabilize the retracted state of Ub.

Structurally, the enhanced sensitivity of the conformational switch for pS65/pT66 Ub can be attributed to the electrostatic interaction between pT66 phosphoryl group and K63 sidechain (Fig. 1G). To confirm this, we introduced a T66E mutation and obtained a pS65 Ub/T66E mutant. The ^31^P NMR spectrum of pS65 phosphoryl group of the protein also shows two peaks, indicating that this mutant protein exists in two slowly interconverting conformational states, i.e. C-terminally relaxed and retracted states. The chemical shift values of the two ^31^P peaks also vary with the pH (Fig. S10A), yielding disparate pKa values for pS65 phosphoryl group in the two conformational states (Fig. S10B). Importantly, the population of the retracted state, as assessed by the volume of the downfield ^31^P peak, reaches ~80% at slightly basic pH (Fig. S10C), and exhibits a similar profile to that of the pS65/pT66 Ub. Thus, the T66E residue can mimic deprotonated pT66 phosphoryl group, and the electrostatic interaction between T66E and K63 sidechains can also stabilize the retracted state. On the other hand, the population of the relaxed state, as assessed by the volume of the upfield ^31^P peak, is less than 60% at acidic pH. Thus, at this pH range, the T66E mutation failed to mimic the phosphoryl group. This is because the glutamate sidechain has a pKa value below 5, and as a result, the relaxed-state structure is destabilized by the unfavorably electrostatic interaction between pS65 phosphoryl group and T66E sidechain. Taken together, the double-phosphorylated Ub is protonated in the relaxed state, with fewer negative charges than the retracted state (Fig. 2G and 2H).

In conclusion, we have shown that PINK1 can phosphorylate Ub at both S65 and T66 residues. There have been several structural studies for the structural basis for S65 phosphorylation by PINK1 (Fig. 2I) (Okatsu et al., 2018). However, it has been shown that Ub binds to PINK1 with a *K*_D_ value of much greater than 300 µmol/L (Gladkova et al., 2017). As weak binding usually corresponds to dynamic interactions (Liu et al., 2016), it is likely that Ub binds to PINK1 in a slightly different pose, thus allowing T66 phosphorylation (Fig. 2J). In the present study, we report that the additional pT66 phosphoryl group enhances the pH-sensitivity of Ub conformational switch, and the multisite phosphorylation makes Ub a better pH sensor. Thus, our study here illustrates a new functional aspect of multisite phosphorylation. It is known that the intracellular pH can vary from >8 inside mitochondria to <7 in Golgi and other membraned organelles. Inflammation, aging, tumorigenesis, apoptosis, autophagy and many other pathophysiological conditions can all cause changes to mitochondrial and cytosolic pH (Berezhnov et al., 2016). Significantly, an increased and slightly basic intracellular pH has been recognized as a hallmark of cancer cells (White et al., 2017). Nevertheless, pH-sensing has been traditionally attributed to histidine sidechains. Our findings here suggest that, the increased pH in cells can promote the conformational switch of the double-phosphorylated Ub to the retracted state, thus eliciting downstream signals.

## Electronic supplementary material

Below is the link to the electronic supplementary material.

Supplementary material 1 (PDF 1907 kb)

## References

[CR1] Bah A, Vernon RM, Siddiqui Z, Krzeminski M, Muhandiram R, Zhao C, Sonenberg N, Kay LE, Forman-Kay JD (2015). Folding of an intrinsically disordered protein by phosphorylation as a regulatory switch. Nature.

[CR2] Berezhnov AV, Soutar MP, Fedotova EI, Frolova MS, Plun-Favreau H, Zinchenko VP, Abramov AY (2016). Intracellular pH modulates autophagy and mitophagy. J Biol Chem.

[CR3] Bienkiewicz EA, Lumb KJ (1999). Random-coil chemical shifts of phosphorylated amino acids. J Biomol NMR.

[CR4] Dong X, Gong Z, Lu Y-B, Liu K, Qin L-Y, Ran M-L, Zhang C-L, Liu Z, Zhang W-P, Tang C (2017). Ubiquitin S65 phosphorylation engenders a pH-sensitive conformational switch. Proc Natl Acad Sci USA.

[CR5] Gao J, Li M, Qin S, Zhang T, Jiang S, Hu Y, Deng Y, Zhang C, You D, Li H (2016). Cytosolic PINK1 promotes the targeting of ubiquitinated proteins to the aggresome-autophagy pathway during proteasomal stress. Autophagy.

[CR6] Gladkova C, Schubert AF, Wagstaff JL, Pruneda JN, Freund SM, Komander D (2017). An invisible ubiquitin conformation is required for efficient phosphorylation by PINK1. EMBO J.

[CR7] Gladkova C, Maslen SL, Skehel JM, Komander D (2018). Mechanism of parkin activation by PINK1. Nature.

[CR8] Kane LA, Lazarou M, Fogel AI, Li Y, Yamano K, Sarraf SA, Banerjee S, Youle RJ (2014). PINK1 phosphorylates ubiquitin to activate Parkin E3 ubiquitin ligase activity. J Cell Biol.

[CR9] Kapuy O, Barik D, Sananes MR, Tyson JJ, Novak B (2009). Bistability by multiple phosphorylation of regulatory proteins. Prog Biophys Mol Biol.

[CR10] Koyano F, Okatsu K, Kosako H, Tamura Y, Go E, Kimura M, Kimura Y, Tsuchiya H, Yoshihara H, Hirokawa T (2014). Ubiquitin is phosphorylated by PINK1 to activate parkin. Nature.

[CR11] Liu Z, Gong Z, Dong X, Tang C (2016). Transient protein-protein interactions visualized by solution NMR. Biochim Biophys Acta Proteins Proteomics.

[CR12] Okatsu K, Sato Y, Yamano K, Matsuda N, Negishi L, Takahashi A, Yamagata A, Goto-Ito S, Mishima M, Ito Y (2018). Structural insights into ubiquitin phosphorylation by PINK1. Sci Rep.

[CR13] Schoppa NE, McCormack K, Tanouye MA, Sigworth FJ (1992). The size of gating charge in wild-type and mutant Shaker potassium channels. Science.

[CR14] Swatek KN, Komander D (2016). Ubiquitin modifications. Cell Res.

[CR15] White KA, Grillo-Hill BK, Barber DL (2017). Cancer cell behaviors mediated by dysregulated pH dynamics at a glance. J Cell Sci.

