# Supplementary material for: Ubiquitin is double-phosphorylated by PINK1 for enhanced pH-sensitivity of conformational switch
Source: Protein Cell. 2019 Jul 5;10(12):908–13. doi: 10.1007/s13238-019-0644-x (PMC6881470; doi:10.1007/s13238-019-0644-x)
Supplement: Supplementary file 1 — Supplementary material 1 (PDF 1907 kb) [file 13238_2019_644_MOESM1_ESM.pdf]

Supplementary File for

Ubiquitin is double-phosphorylated by PINK1 for enhanced pH-sensitivity of conformational switch

Shang-Xiang Ye, Zhou Gong, Ju Yang, Yu-Xin An, Zhu Liu, Qun Zhao, Ewen Lescop, Xu Dong*, and Chun Tang*

Abstract: A signaling protein can be phosphorylated at multiple sites to gain new function. Ubiquitin (Ub), an important signaling protein in cells, can be phosphorylated by kinase PINK1 at residue S65, and the resulting pS65 Ub was shown to adopt two alternative conformations. Here we report that PINK1 can also phosphorylate Ub at residue T66. Though T66 phosphorylation *per se* does not perturb Ub structure, pS65/pT66 Ub interconverts between C-terminally relaxed- and retracted-state conformations in response to subtle pH change. Comparing to pS65 Ub, the conformational switch of the double phosphorylated Ub has an enhanced pH sensitivity, and the retracted state is more populated at slightly basic pH. Further we show that, the pH sensitivity of Ub conformational switch is resulted from disparate pKa values of the phosphoryl groups in the two conformational states, and the retracted state is stabilized by the additional T66 phosphoryl group. Thus, the multisite phosphorylation can make Ub a better pH sensor, translating pH variation into corresponding cell signals.

Methods

Sample preparation and mass spectrometry analysis. Human ubiquitin was cloned to a pET11a vector. S65A and T66E point mutations were introduced using QuikChange method. BL21 star cells were used for protein expression, and were grown in either LB medium (for preparing unlabeled proteins) or in M9-minimal medium (for preparing isotope-enriched proteins). Ubiquitin proteins were purified through Sepharose SP and Sephacryl S100 columns (GE Healthcare) in tandem. For isotope enrichment, 1 g/L U-^{15}N–labeled NH_4Cl (Isotec) and/or 2 g/L U-^{13}C-labeled glucose (Isotec) were added to *Escherichia coli* culture as the sole nitrogen and/or carbon source.

PINK1 from body louse (*Ph*PINK1), pS65 Ub/T66E and pUb were prepared as previously described[1]. To prepare double phosphorylated ubiquitin and pT66 Ub/S65A, PINK1, Ub (or S65A Ub), $MgCl_2$, and ATP were mixed at the molar ratio of 1:10:500:500 in 20 mM MES buffer (pH 6.4, containing 1 mM DTT). The reaction proceeded at room temperature overnight. The produced ppUb was purified with Superdex-75 and Source Q columns (GE Healthcare). The overall mass of the protein, with the extra mass for one or two phosphoryl groups, was confirmed by ESI mass spectrometry (Bruker Daltonics). To identify ubiquitin phosphorylation sites, ubiquitin sample was subjected to trypsin digestion. The resulting peptide mixture was subjected to targeted LC-MS/MS on a Easy1000-LC Orbitrap QE-nano-spray (Thermo Fisher Scientific). Data was analyzed using Xcalibur 2.2 SP1.48.

HEK293 cells were purchased from the Institute of Cell Biology of the Chinese Academy of Sciences (Cell Biology of the Chinese Academy of Sciences, Shanghai, China). Cells were grown in Dulbecco's modified essential medium (DMEM, Gibco by Thermo-Fisher Scientific) supplemented with 10% heat-inactivated fetal bovine serum (Zhejiang Tianhang Biotechnology, China) at 37 ℃, in the atmosphere of 5% CO_2. Cells were co-transfected with soluble human PINK1 (residues L102-L521, with the N-terminal mitochondrial targeting sequence removed[2]) and Ub 7KR (mutating all seven lysines to arginines to prevent the formation of polyubiquitin) with polyethyleneimine (PEI, linear with average molecular weight 25,000 Da, Alfa-Aesar Chemical, Shanghai, China). The medium containing the transfection reagents was removed and fresh medium was added at 6 h after transfection. 36 hours after transfection, the cells were washed with PBS buffer, and the cells were lysed for 60 min at 4 ℃ in lysis buffer.

The proteins extracted from HEK293 cells were subjected to trypsin digestion. The resulting peptide mixture was subjected to an Easy-nLC 1200 system coupled to an Orbitrap Fusion Lumos mass spectrometer (Thermo Fisher Scientific, San Jose, CA, USA). Samples were automatically loaded onto a C18 RP-trap column (150 μm i.d. x 3 cm) and separated by a C18 capillary column (150 μm i.d. x 15 cm), packed in-house with ReproSil-Pur C18-AQ particles (1.9 μm, 120 Å). Mobile phase A is 0.1% FA in HPLC H_2O, and mobile phase B is 80% acetonitrile, 0.1% FA and 20% HPLC H_2O. The column was run with a 75-min gradient at the flow rate of 600 nL/min. The mass spectrometer was operated in the data-dependent mode with one full MS scan at R = 60, 000 (m/z = 200), followed by HCD MS/MS scans at R = 15, 000 (m/z = 200), NCE = 30, with an isolation width of 1.6 m/z. The AGC targets for the MS1 and MS2 scan were 4e+5 and 5e+4, respectively, and the maximum injection times for MS1 and MS2 were both 50 ms. Only precursors with charge states 2 to 7 and with an intensity higher than 20, 000 were selected for fragmentation, with the dynamic exclusion set to 20 s. The pFind Studio 3 software[3] was used to identify peptides derived from pS65/pT66 Ub. The search parameters were set as following: instrument HCD, precursor mass tolerance 20 ppm, and fragment mass tolerance 20 ppm, with full tryptic cleavage constraints but allowing up to 3 missed cleavages.

Ubiquitin sequence, either wildtype or 7KR mutant, was included in the library, with a spectral false identification rate ≤ 1%.

NMR data acquisition for pS65/pT66 Ub. The protein sample was prepared in 20 mM HEPES buffer (150 mM NaCl and pH 7.4, with 5% D_2O). NMR experiments were recorded at 25 ℃ on Bruker 600 MHz and 850 MHz spectrometers, both equipped with cryoprobes. ^1H-^{15}N HSQC, HNCA, HNCB, CCOHN, HCCONH, HCCH-TOCSY and HNCO data were collected to confirm the assignment of backbone and side chain resonances. ^{15}N-edited and ^{13}C-edited NOESY experiments were acquired with a 120 ms mixing time for NOE distance restraints. Residual dipolar couplings (RDC) was recorded for backbone amide bond vectors in PEG (C12E5)/hexanol (6%; Sigma-Aldrich), PEG (C12E5)/hexanol/CTAB (6%, PEG:CTAB ratio 30:1; Sigma-Aldrich) and (C12E5)/hexanol/SDS (6%, PEG:SDS molar ratio 30:1; Sigma-Aldrich), using the in-phase/anti-phase scheme of HSQC. The NMR data were processed using NMRPipe,[4] and NOE assignment was performed with CCPNmr Analysis.[5] The chemical shift perturbations (CSPs) were calculated with $[0.5\times\Delta\delta H^2+0.1\times\Delta\delta N^2)]^{0.5}$, in which $\Delta\delta H$ and $\Delta\delta N$ were the chemical shift difference in ^1H and ^{15}N dimensions, respectively.

The ^{31}P 1D spectra were recorded on a Bruker 500-MHz spectrometer equipped with a BBO nitrogen-cooled probe. The ^{31}P base frequency is 202.456 MHz, with 0 ppm referenced to 10% phosphoric acid. Protons were decoupled with WALTZ16 during ^{31}P channel acquisition. The four ^{31}P peaks were assigned based on these two observations: first, the chemical shift values for serine phosphoryl group is more downfield than that of threonine phosphoryl group,[6] and second, the chemical shift values of protonated phosphoryl group is more downfield and deviates from literature values. The peak volumes in 1D NMR spectra were integrated with Bruker TopSpin Version 3.5.

NMR pH titration. The initial NMR sample were in 20 mM HEPES buffer (150 mM NaCl and pH 7.4), and the protein solution was transferred from the NMR tube into a 1.5 ml Eppendorf tube for pH adjustment using a Micro Combination pH electrode (Mettler Toledo). A series of ^1H-^{15}N HSQC spectra were recorded for the pS65/pT66 Ub sample at pH 5.0, pH 5.6, pH 6.2, pH 6.8 and pH 7.4 using a Bruker 600MHz NMR spectrometer. The signal intensities relating to the resonances of the retracted state and relaxed state of pS65/pT66 Ub were taken to determined their population under different pH conditions.

For ^{31}P pH titrations, the sample was prepared as the HSQC titration except the pH range. The pH titration for ^{31}P experiment was taken from pH 5.0 to pH 8.6 with 0.6 pH-unit increment per titration. The ^{31}P 1D spectra were recorded for pS65/pT66 Ub, pT66 Ub/S65A and pS65 Ub/T66E on a Bruker 500-MHz spectrometer equipped with a BBO cryoprobe. The ^{31}P chemical shift values for relaxed and retracted states as a function of pH was fitted to an acid–base equilibrium. The population change upon pH titration was fitted with Boltzmann sigmoid function,

$$Population_{relaxed} = Population_{max} - \frac{Population_{max} - Population_{min}}{1 + \exp(\frac{pH_{50} - pH}{slope})}$$

$$Population_{retracted} = Population_{min} + \frac{Population_{max} - Population_{min}}{1 + \exp(\frac{pH_{50} - pH}{slope})}$$

Here pH_{50} is the pH value when the two conformational states are equally populated, and the slope describes the steepness of the transition. The Boltzmann sigmoid function has been used to describe the coupling between movement of potassium ions and conformational change of voltage-gated ion channels, in which the slope is proportional to the total charge displaced.[7]

Protein structure calculation. The structures of relaxed and retracted state of pS65/pT66 Ub were calculated separately using Xplor-NIH[8]. The topology and parameter files for phosphorylated serine (SEP) and threonine (TPO) were generated with PRODRG server[9]. The φ and ψ dihedral angle restraints were analyzed and generated using TALOS+[10] from backbone chemical shift values. The structures were refined against NOE distance restraints, TALOS dihedral angle restraints and RDC restraints collected in three alignment media with addition of weak radius-of-gyration restraint[11] and knowledge-based database restraint.[12] 240 structures each were calculated for relaxed states and retracted state of pS65/pT66 Ub. The top 20 structures with lowest energy were selected for structural analysis. The quality of the calculated structures was evaluated using WHATCHECK[13] and PROCHECK[14]. Structure figures were rendered using PyMOL Version 2.2 (Schrödinger).

Stability measurements of Ub, pS65 Ub and pS65/pT66 Ub. Differential scanning fluorimetry (DSF) was performed using the Prometheus NT.48 instrument (NanoTemper Technologies, GmbH). Samples of Ub, pS65 Ub and pS65/pT66 Ub were prepared in 20 mM HEPES, 150 mM NaCl, pH 7.4 buffer. The temperature was increased with a heating rate of 1℃ /min from 25℃ to 100℃, and the fluorescence at emission wavelengths of 330 nm and 350 nm was measured. The T_m value was obtained using the progress function in the software supplied with the instrument. Transition regions of unfolding profiles were fitted using the Boltzmann equation to determine T_m.

MD simulations and protein stability analysis. MD simulations were performed using AMBER 16 package with ff14SB forcefield.[15] The starting conformations for relaxed and retracted state are the conformers closest to the mean in NMR structure bundle calculated. The parameters for SEP and TPO in various protonated states were taken from Ref. [16]. The structure was solvated in a cube containing TIP3P water molecules, with at least 10 Å padding in all direction, and was energy minimized. MD simulation trajectory of 200 ns was recorded with 2000 snapshots at 100 ps interval.

We have used two approaches to characterize the unfolding process of proteins, either by heating the system to 373K or by placing the protein in 8M urea. In the latter approach, the periodic solvent box has the mixture of 8M urea and water molecules. The box is pre-equilibrated at 300K and 1 atm for 0.5 ns, and the RESP charges were used for urea molecules.[17] The analyses were performed using PTRAJ module in AMBER. The RMS deviations (RMSD) and RMS fluctuations (RMSF) were calculated for the whole simulation trajectories for Cα atoms of residues 1 to 72. The free energy landscape for the unfolding process were defined as $-K_B RT ln(\frac{NR}{NT})$, where NR is the number of conformations in each bin (0.1 Å defined by the RMSD), NT is the total number of the conformations, K_B is the Boltzmann constant, and T is 298K.

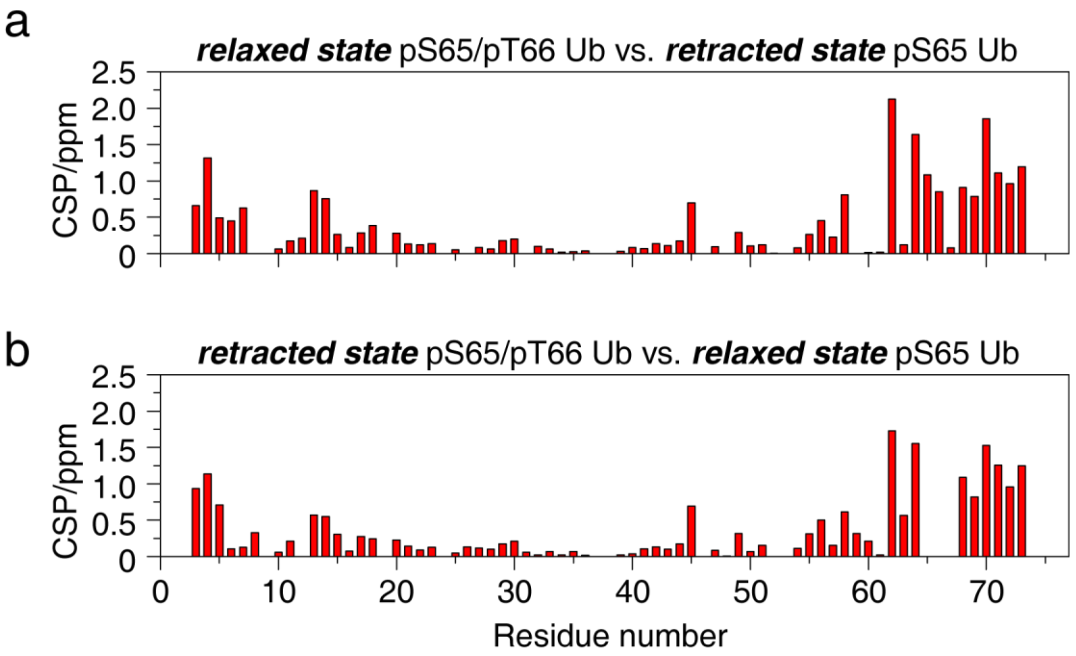

Figure S1. NMR classification of C-terminally relaxed and retracted states of pS65/pT66 Ub. The chemical shift perturbations (CSPs) are large, when (a) pS65/pT66 Ub relaxed state is compared to pS65 Ub retracted state, and (b) pS65/pT66 Ub retracted state is compared to pS65 Ub relaxed state. The averaged CSP in ppm is calculated with the equation $\Delta\delta=[0.5\times\Delta\delta H^2+0.1\times\Delta\delta N^2)]^{1/2}$, with ΔδH and ΔδN as the CSPs in ^{1}H and ^{15}N dimensions.

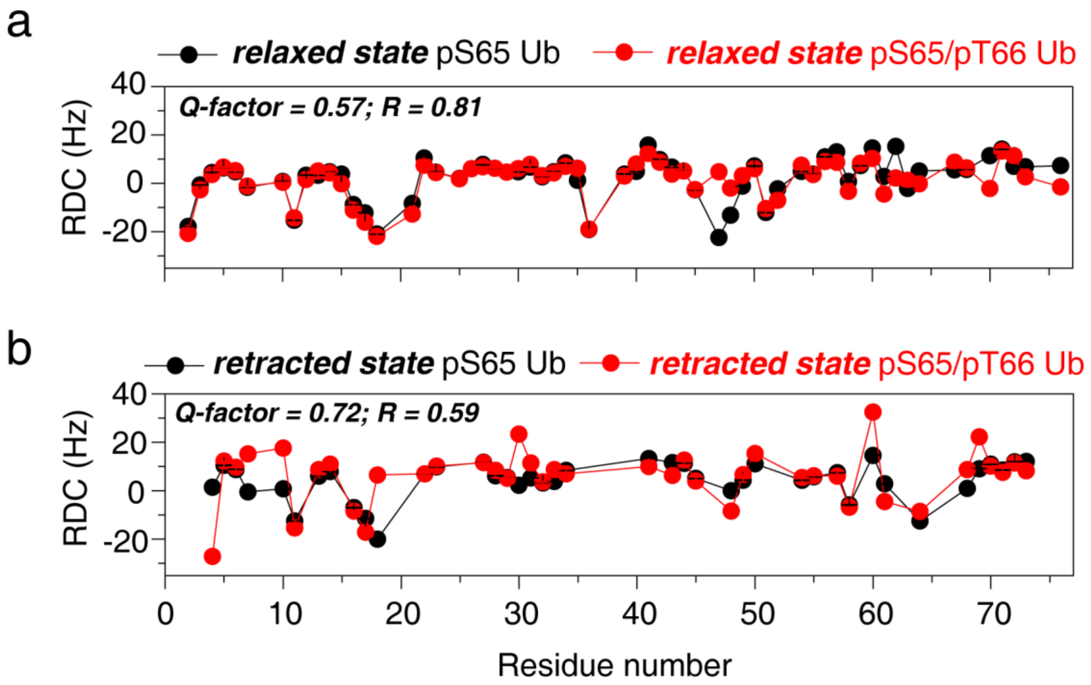

Figure S2. Residual dipolar coupling (RDC) collected in PEG (C12E5)/hexanol, for the backbone NH bond vectors of the relaxed state (a) and retracted state (b) of pS65 Ub and pS65/pT66 Ub. Though the overall RDC profiles look similar, there are regions with large discrepancy. The RDC agreement is better for the relaxed state than for the retracted state, whose R factors are 0.81 and 0.59, respectively. The RDC Q-factor for the relaxed state and retracted state are 0.57 and 0.72, respectively.

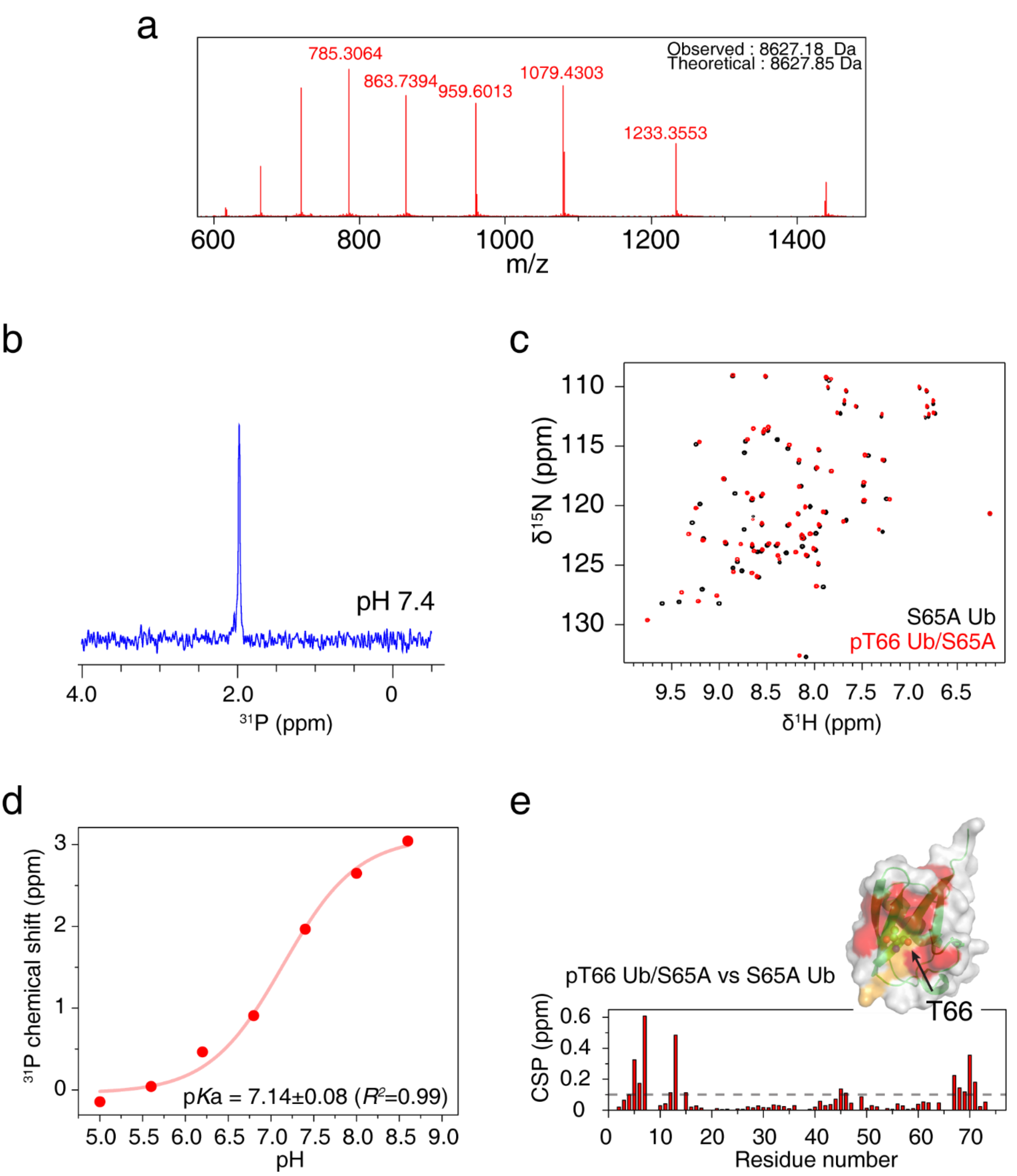

Figure S3. Structural property of pT66 Ub/S65A Ub. (a) MS analysis of pT66 Ub/S65A, which shows the phosphorylation of Ub T66 by PINK1 regardless of S65 phosphorylation. (b) The single ^{31}P NMR signal observed for pT66 Ub/S65A at pH 7.4. (c) The 2D ^{1}H-^{15}N HSQC spectrum shows a single set of peaks. (d) Fitting ^{31}P chemical shift values of the single ^{31}P peak, as a function of pH, affords the pKa value of pT66 phosphoryl group. (e) The CSPs between pT66 Ub/S65A and Ub/S65A can be mapped to around pT66, which mainly include residues in β1, β2 and β5. Inset: residues with CSPs > 0.1 ppm (dotted line) are mapped onto the structure of Ub/S65A, and are colored red; unassigned contiguous residues are colored yellow.

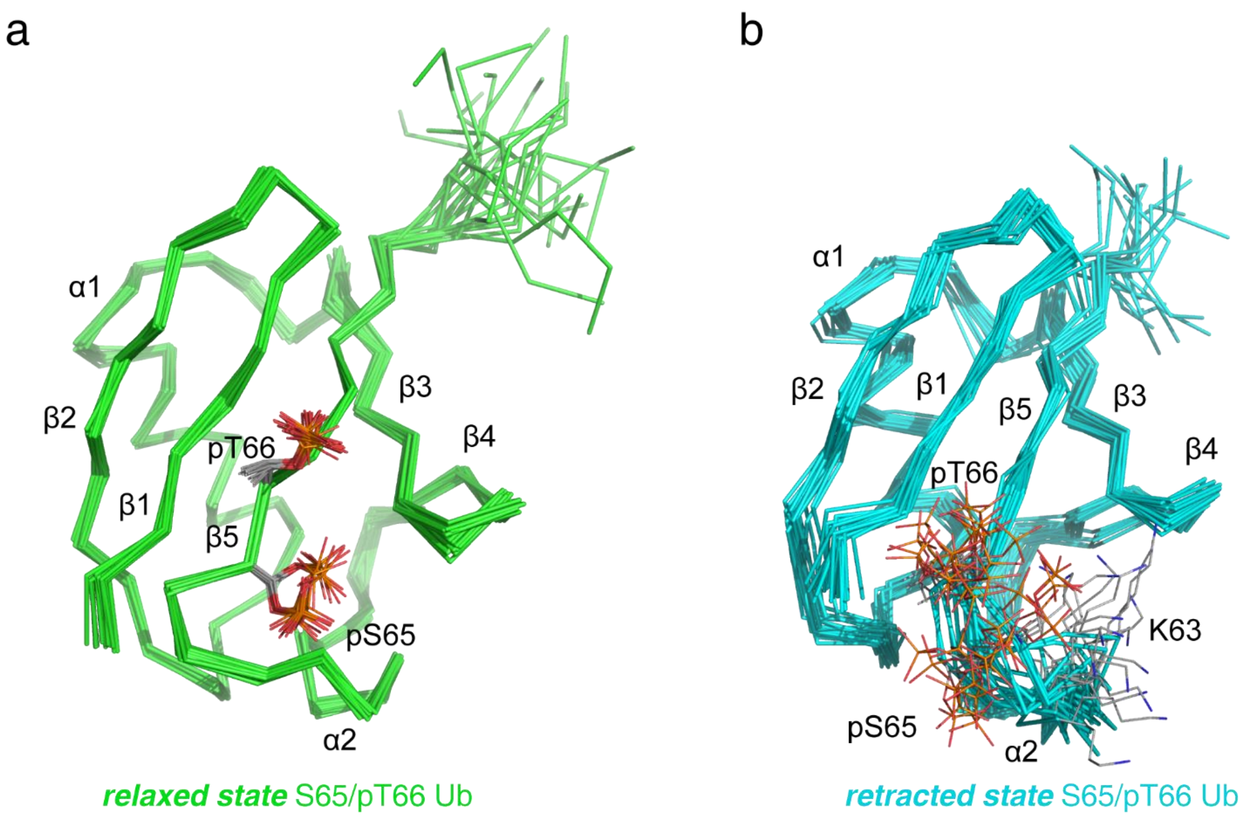

Figure S4. Solution structures of (a) relaxed and (b) retracted states of pS65/pT66 Ub. 20 calculated structures are superimposed by the backbone heavy atoms of residues 1-71 in the relaxed state and residues 1-73 in the retracted state. The RMS deviations are 0.32±0.04 Å for the relaxed state, and 0.75±0.09 Å for the retracted state. The pS65 and pT66 of the relaxed and retracted states, as well as K63 in the retracted state, are shown as grey lines, and the protruding loop preceding β5 of the retracted state is characterized with low convergence.

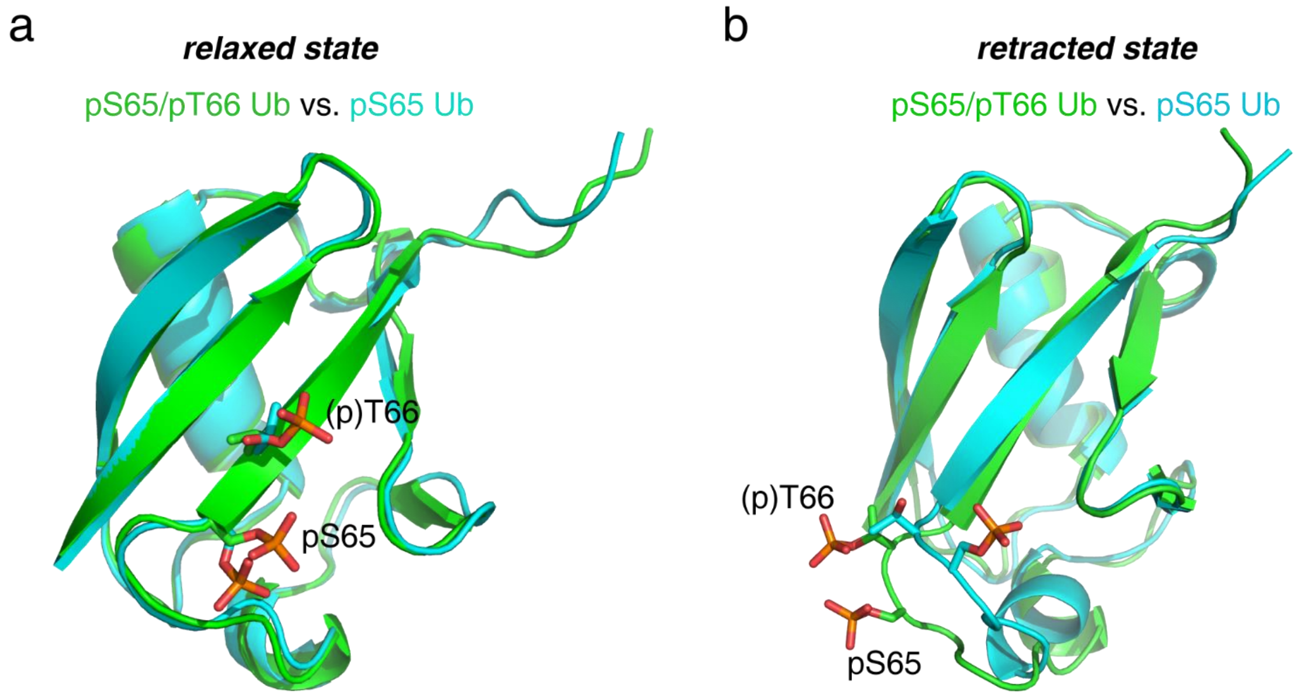

Figure S5. Structural comparison between pS65/pT66 Ub (green) and pS65 Ub (cyan). (a) Comparison of the solution structures of the relaxed state gives an RMS difference of 0.79 ± 0.06 Å for backbone heavy atoms of residues 1-71. (b) Comparison of the solution structure of the retracted state gives an RMS difference of 1.81 ± 0.11 Å for backbone heavy atoms of residues 1-73.

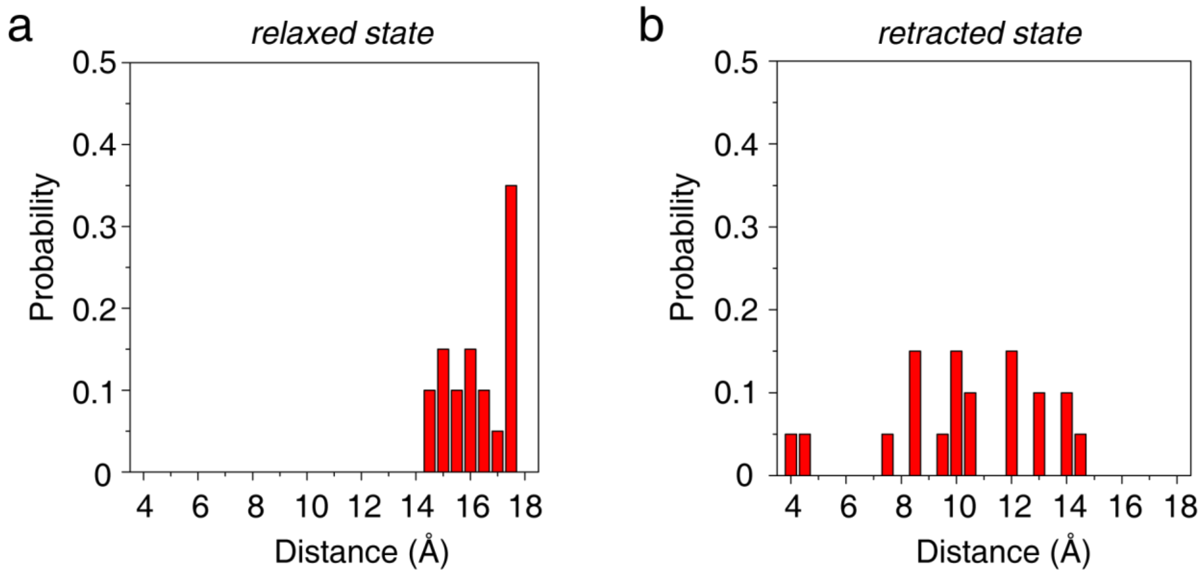

Figure S6. Distribution of the distances between the charged oxygen atoms in pT66 phosphoryl groups and charged nitrogen atom in K63 side chain for the relaxed state (a) and retracted state (b) of pS65/pT66 Ub. Owing to rapid rotation of the phosphoryl groups, the distance is calculated as $<r^{-6}>^{-1/6}$ for the 3 pairs of oxygen-oxygen distances, and categorized in 0.5 Å bins.

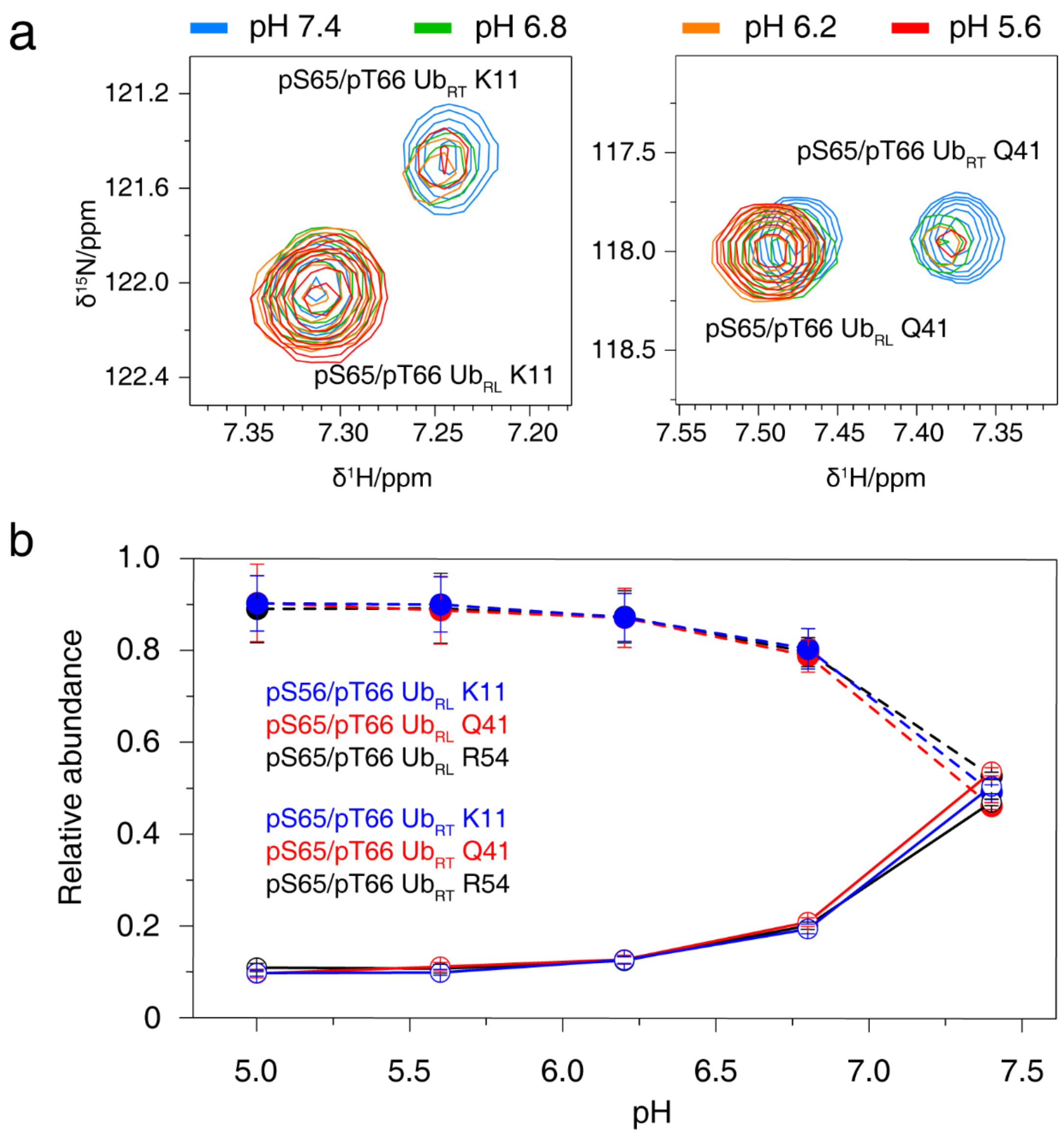

Figure S7. The relaxed state of pS65/pT66 Ub is more populated than the retracted state at acidic pH. (a) Representative regions of a series of 2D ^{1}H-^{15}N HSQC spectra, collected at four different buffer pHs. (b) Relative abundances of relaxed/retracted states, quantitated by the intensities of well isolated peaks in the 2D spectra.

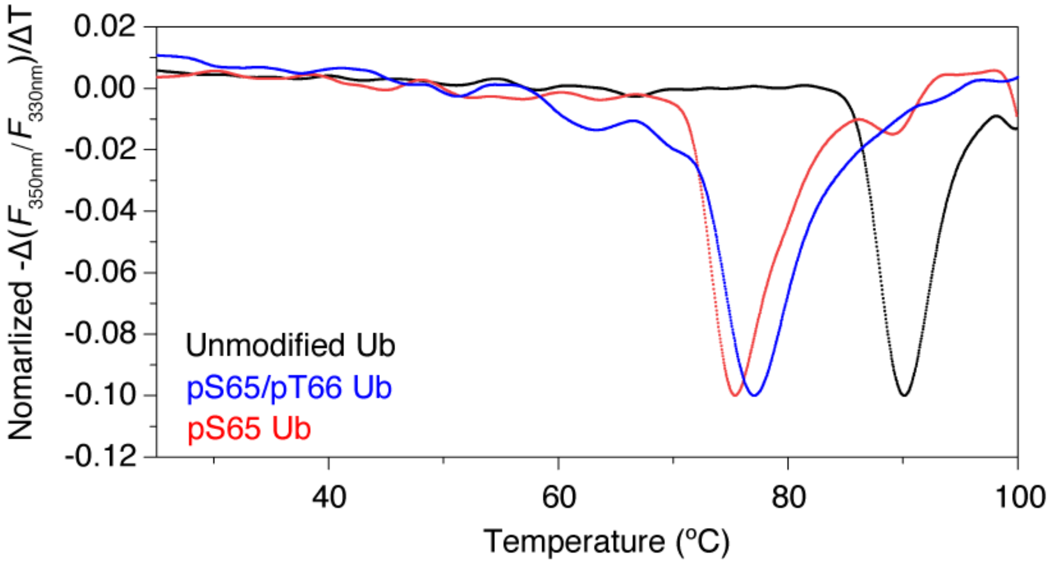

Figure S8. Differential scanning fluorimetry (DSF) analyses of unmodified Ub, pS65 Ub and pS65/pT66 Ub. Single phosphorylation of Ub at S65 decreases the melting temperature (T_m) from 90.3 ℃ to 75.9 ℃. Additional T66 phosphorylation increases the T_m to 77.2 ℃, indicating higher thermal stability.

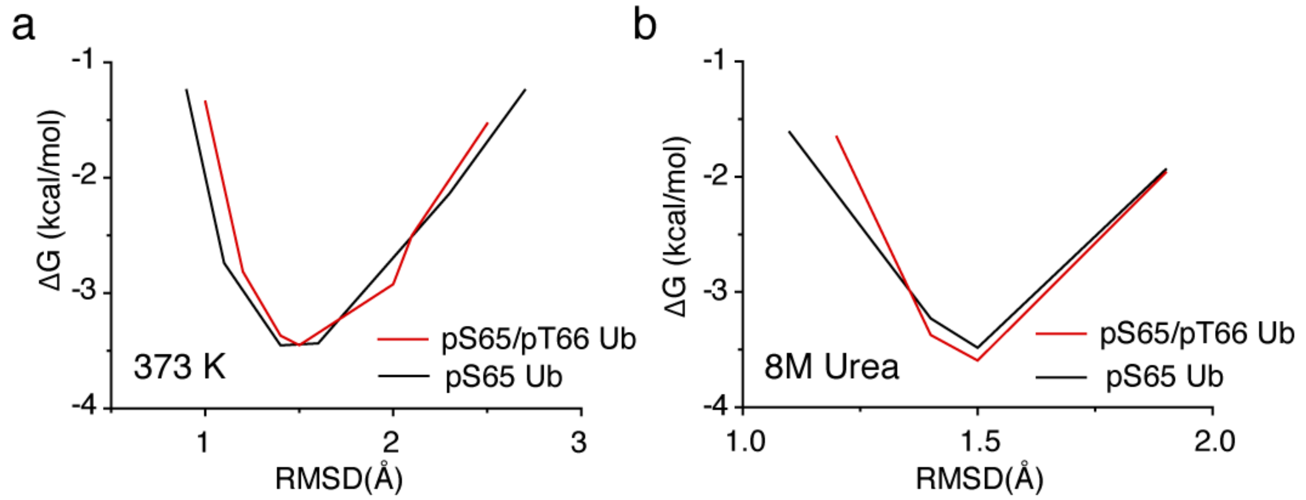

Figure S9. The relaxed states of pS65 Ub and pS65/pT66 Ub have similar stability as assessed by MD simulations. Reconstructed free energy landscape of the unfolding processes of pS65 Ub and pS65/pT66 Ub relaxed states under heat (373 K) (a) and 8 M urea (b) denaturing conditions.

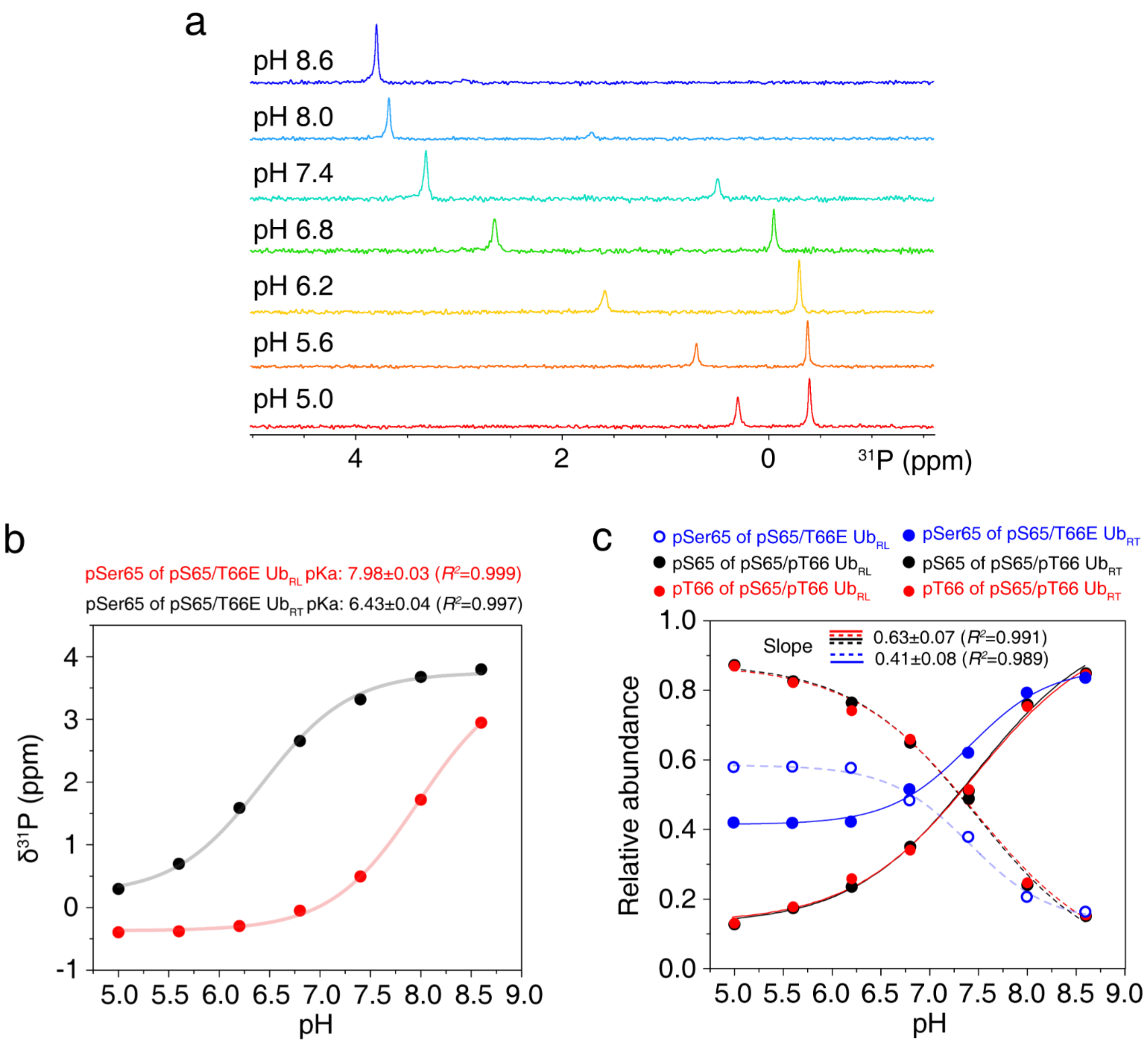

Figure S10. The pH titration of pS65 Ub/T66E is monitored using ^{31}P NMR experiments. (a) The ^{31}P spectra of pS65 Ub/T66E recorded at various pH values. The chemical shifts and intensities of signals change upon pH titration. (b) The pKa of pS65 Ub/T66E obtained by fitting ^{31}P chemical shifts against corresponding pH, and it gives pKa values of 7.98±0.03 and 6.43±0.03 for the relaxed state (Ub_{RL}) and the retracted state (Ub_{RT}). (c) Fitting of the relative abundance of relaxed state (Ub_{RL}) and retracted state (Ub_{RT}) of pS65 Ub/T66E against pH change results in a slope of 0.41±0.08, with R^2 of 0.989.

Table S1 Statistics of 20 calculated structures for pS65/pT66 Ub retracted and relaxed states

	relaxed state[a]	***retracted state***[a]
**Number of restraints**		
NOE	606	529
Intra (i=j)	313	293
Short range (|i-j|<3)	164	143
Long range (|i-j|≥3)	129	93
RDC		
PEG HN	58	40
PEG+SDS HN	53	31
PEG+CTAB HN	56	33
H-bond	50	58
Dihedral angles[b]		
φ/ψ	115	120
**Structure statistics**		
Number of violations (per structure)		
Distance constraints (> 0.5 Å)	0	0
Dihedral angle constraints (> 5º)	0	0
RDC RMS (Hz)	0.66±0.14	0.54±0.10
**Pairwise RMS deviation (Å)**	*residues 1-71*	*residues 1-73*
Back-bone atoms[c]	0.32±0.04	0.75±0.09
All atoms[c]	0.82±0.05	1.33±0.12
**Ramachandran plot**		
most favored	92.3%	81.4%
additionally allowed	5.8%	16.4%
generously allowed	1.1%	0.9%
disallowed	0.8%	1.3%
**Z-scores (residues 1-76)**		
2nd generation packing quality	-1.928	-2.428
Ramachandran plot appearance	-1.330	-3.827
χ^1/χ^2 rotamer normality	-2.386	-1.189
Backbone conformation	1.535	0.041

a: For either the relaxed state or the retracted state, 240 structures were calculated, and 20 structures with lowest energy and least restraint violation were selected. The quality of the selected structures was assessed using PROCHECK[14] and WHATCHECK[13].

b: The dihedral angle restraints were generated using TALOS+[10] from backbone chemical shifts. When 9 out of 10 predictions fall into a consistent region of the Ramachandran plot, the corresponding dihedral angle restraint was used during structure determination.

c: Residues 1-71 of the relaxed state and residues 1-73 of retracted state were superimposed to calculated the RMSD values, respectively.

Supplementary References

[1] X. Dong, Z. Gong, Y. B. Lu, K. Liu, L. Y. Qin, M. L. Ran, C. L. Zhang, Z. Liu, W. P. Zhang, C. Tang, *Proc. Natl. Acad. Sci. U. S. A.* **2017**, *114*, 6770-6775.

[2] J. Gao, M. Li, S. Qin, T. Zhang, S. Jiang, Y. Hu, Y. Deng, C. Zhang, D. You, H. Li, D. Mu, Z. Zhang, C. Jiang, *Autophagy* **2016**, *12*, 632-647.

[3] H. Chi, C. Liu, H. Yang, W. F. Zeng, L. Wu, W. J. Zhou, R. M. Wang, X. N. Niu, Y. H. Ding, Y. Zhang, Z. W. Wang, Z. L. Chen, R. X. Sun, T. Liu, G. M. Tan, M. Q. Dong, P. Xu, P. H. Zhang, S. M. He, *Nat. Biotechnol.* **2018**.

[4] F. Delaglio, S. Grzesiek, G. W. Vuister, G. Zhu, J. Pfeifer, A. Bax, *J. Biomol. NMR* **1995**, *6*, 277-293.

[5] W. F. Vranken, W. Boucher, T. J. Stevens, R. H. Fogh, A. Pajon, M. Llinas, E. L. Ulrich, J. L. Markley, J. Ionides, E. D. Laue, *Proteins* **2005**, *59*, 687-696.

[6] E. A. Bienkiewicz, K. J. Lumb, *J. Biomol. NMR* **1999**, *15*, 203-206.

[7] N. E. Schoppa, K. McCormack, M. A. Tanouye, F. J. Sigworth, *Science* **1992**, *255*, 1712-1715; bZ. Lu, A. M. Klem, Y. Ramu, *J. Gen. Physiol.* **2002**, *120*, 663-676.

[8] C. D. Schwieters, G. A. Bermejo, G. M. Clore, *Protein Sci.* **2018**, *27*, 26-40.

[9] A. W. Schuttelkopf, D. M. van Aalten, *Acta Crystallogr. D Biol. Crystallogr.* **2004**, *60*, 1355-1363.

[10] Y. Shen, F. Delaglio, G. Cornilescu, A. Bax, *J. Biomol. NMR* **2009**, *44*, 213-223.

[11] C. D. Schwieters, G. M. Clore, *J. Phys. Chem. B* **2008**, *112*, 6070-6073.

[12] G. A. Bermejo, G. M. Clore, C. D. Schwieters, *Protein Sci.* **2012**, *21*, 1824-1836.

[13] R. W. Hooft, G. Vriend, C. Sander, E. E. Abola, *Nature* **1996**, *381*, 272.

[14] R. A. Laskowski, M. W. Macarthur, D. S. Moss, J. M. Thornton, *J. Appl. Crystallogr.* **1993**, *26*, 283-291.

[15] J. A. Maier, C. Martinez, K. Kasavajhala, L. Wickstrom, K. E. Hauser, C. Simmerling, *J. Chem. Theory Comput.* **2015**, *11*, 3696-3713.

[16] N. Homeyer, A. H. Horn, H. Lanig, H. Sticht, *J. Mol. Model* **2006**, *12*, 281-289.

[17] C. I. Bayly, P. Cieplak, W. D. Cornell, P. A. Kollman, *J. Phys. Chem.* **1993**, *97*, 10269-10280.
